# Coordination of cybersecurity risk management in the U.K. insurance sector

**DOI:** 10.1057/s41288-023-00287-9

**Published:** 2023-02-10

**Authors:** Paul Klumpes

**Affiliations:** Aalborg University Business School, Aalborg University, Fiberstraede 2-41, 9200 Aalborg Ost, Denmark

**Keywords:** Coordination, Cyberattack, Regulators, U.K.

## Abstract

The increasing threat of cyberattacks has resulted in increased efforts by both the U.K. government and regulatory authorities to coordinate efforts to influence cybersecurity risk management practices in the U.K. insurance sector, focusing on cyber risk underwriters. This paper provides an evaluation of these arrangements. It first provides a descriptive overview of the key U.K. regulatory authorities and the evolution of their efforts over the past decade, as well as the scope for broader collaborations with industry and member-based associations and international organisations. It then evaluates the effectiveness of these efforts by providing a multi-method study of the incidence, nature and evolution of cost of data breaches, investment in computer systems and software intangible assets at risk of cyberattack, and a content analysis of annual reports of both U.K. regulators and a sample of U.K. insurers. The findings suggest that while both the total costs of data breaches and the size of investment in computer systems and software intangibles at risk of cyberattack have gradually increased over time, the degree of engagement with cyber as a reporting issue by both cyber insurers and financial regulators has not. It is concluded that while these efforts have been apparently successful in avoiding a large-scale, systemic cyberattack on the U.K. insurance industry, there are significant gaps and overlaps in the system of cyber regulatory oversight.

## Introduction

London is currently the world’s leading financial centre within the increasingly integrated, technologically sophisticated and growing global financial system. Moreover, the U.K. financial services sector provides a significant contribution to the overall wealth of the U.K. and is therefore a key element of the nation’s Critical National Infrastructure (CNI).^1^ Responsible financial service entities operating in the U.K. have become increasingly sensitive to and concerned about cybersecurity risk in recent years.^2^ It is therefore important to develop more integrated and timely monitoring systems that effectively communicate the associated information risk from the IT and operational risk areas to the board. However, there are currently still no U.K. regulations that specifically address either the appropriate protocols for networks to mitigate against these threats, or the reporting of such risks to the board, regulators and key stakeholders.^3^ There are also internal governance implications. Dutta and McCrohan (2002) argue that cybersecurity risk management is a management and not an IT issue. However, these two issues have not been previously studied in a single paper.

Key U.K. insurance firms trade off the benefits of enhancing their business model through exploiting developments in cloud computing and big data with the costs of investing in cyber risk management, and the strategies employed (e.g. via insurance, regulatory compliance and operational management). However, these efforts are subject to dynamic and increasingly integrated cyber threats from various sources. First, there are evolving frictional risks from increased direct and hidden costs of complying with EU and U.K. data protection laws (Grady and Parisi 2006). Second, there is significant and material investment in cybersecurity resilience-based audit and IT departments. Consultants offer competitive and new digital security insurance and risk management solutions across the sector and best practices used by key insurance firms to identify fraud losses and potential theft of personal data held by organisations, related to both internal and external parties. Third, the rapid growth of information technology-based solutions has facilitated the globalisation of services and transformed business models, which in turn has resulted in growing demand for cyber insurance. However, public awareness of the increasingly systemic nature of cyber risk has also been growing in recent years, particularly following the COVID-19 pandemic and related increase in the incidence of cyberattacks as organisations imposed home-working practices on their employees. Finally, the increasing incidence of systemic cyberattacks following Russia’s invasion of Ukraine in early 2022 has caused underwriters to limit their exposure, such as by increasing prices and amending policies to ensure that policyholders retain more losses (Smith 2022).^4^

U.K. cyber underwriting insurance firms are particularly susceptible to cybersecurity attacks because of the double materiality nature of their business models, which involves interconnected responsibilities for maintaining the resilience of their systems to various gatekeepers (e.g. regulatory agencies), actors (e.g. other financial services entities) and stakeholders (e.g. shareholders, consumers), which create pressures to ensure best practices in information risk governance, data and information management. To achieve success in international markets, U.K.-based global cyber insurance policy providers also face challenges of moving towards competing on being able to offer unique, high-quality cyber assurance and innovative integration solutions to their financial services clients.

A key challenge facing such firms is to demonstrate sufficient ethical management, and ensure high-quality data integrity capabilities in order to meet increasingly stringent and complex requirements imposed by regulators. This, however, also requires firms to face the need to trade off investment in high-quality regulatory compliance monitoring mechanisms, with providing high-quality, value-added services and performance to their clients and investors, respectively. However, the financial services industry generally, and the insurance industry specifically, particularly in globally exposed markets such as the U.K., face unique challenges in a changing regulatory environment. Further, the implementation of Solvency II capital adequacy requirements in 2016 required insurance companies to rely even more on complex financial models that require integration with existing financial, regulatory and customer databases. Enhanced infrastructure protection through cyber risk insurance is therefore a key concern for financial service firms and their stakeholders.

However, although quality investment in cybersecurity risk management coordination processes is essential to meet regulatory needs and enhance the robustness and integrity of financial services firms’ data and information exchange, its impact on key external stakeholders and gatekeepers has not been previously systematically studied.^5^ There has also historically been a lack of public information sharing and engagement about these issues among U.K.-regulated cyber insurance firms and financial regulatory organisations.^6^

However, there is a lack of prior literature evidencing the effectiveness of such coordination efforts to mitigate or prevent cyberattacks. There is little empirical, conceptual or analytical academic research specifically on cyber risk and/or cyber insurance that is of relevance to the research questions that assess the quality of cyber risk coordination efforts. In the insurance sector context, Eling (2020) calls for more research on information sharing between public and private actors, in the form of public–private partnerships. Shackleford (2011) argues that firms should adopt an initiative-taking approach to safeguard their assets against attack in a competitive environment. Biener et al. (2015) provide evidence of the insurability of cyber risk in a European and U.S. context and find that there are significant problems in the market due to adverse selection, resulting from highly inter-correlated losses, lack of data and severe information asymmetry. They also provide evidence that there is a distinct lack of cyber insurance coverage available in the European context, in contrast to the U.S., due to the lack of public policy engagement and reluctance of firms to disclose breaches. This situation contrasts with that in the U.S., where the Securities and Exchange Commission has issued guidance on the disclosure of security breaches by U.S. corporations. This in turn has facilitated empirical studies on the effectiveness of such disclosure requirements (e.g. Dutta and McCrohan 2002; Wang et al. 2013; Ferraro 2014).

This paper fills the gap in the literature by examining the recent evolution of efforts by U.K. regulators, both domestically and more broadly in collaboration with international bodies, to coordinate with the U.K. cyber insurance industry to enhance its resilience against cyberattacks. It also informs the business and academic community generally about the nature and outcomes of regulatory efforts to coordinate the resilience of insurance firms against cyber risk. The overall research objective is to address calls for more evidence-based framing strategies to help increase societal and political awareness of cybersecurity risk management coordination issues (de Bruijn and Janssen 2017). This paper first overviews the scale and scope of these efforts over the last decade, and then undertakes an evaluation of the effectiveness of these efforts by both U.K. cyber insurance companies and their U.K. regulators to assure the resilience of their systems against cyberattacks. This research has also significant public policy relevance, given the increasing societal-wide public concerns about the integrity and resilience of these resources to withstand increasingly state-based systemic cyber risk.^7^

Three research questions are addressed:

1. What is the nature and evolution of regulatory efforts to coordinate cybersecurity risk management of the U.K. cyber insurance industry since 2014?
2. What is the scale and scope of these efforts, both in terms of the extent of coordination efforts and the degree of collaboration with other regulators, industry bodies, membership organisations and internationally?
3. Have these regulatory coordination efforts been effective in mitigating cybersecurity risk exposure of both U.K. cyber insurance firms and the regulatory bodies involved?

The rest of this paper is organised as follows. The next section addresses the first research question by providing an overview of the evolution of regulatory interventions related to cybersecurity risk management in the U.K. insurance industry. The subsequent section addresses the second research question by evaluating the scale and scope of collaborative efforts nationally between key U.K. regulators and other industry organisations. The paper then examines the role of U.K. regulators in cybersecurity coordination efforts at the international level. The penultimate section undertakes a multi-method approach to examine the nature, incidence and evolution of cyber risk exposures, in terms of size, nature and transparency of (cyber and non-cyber) specialist U.K.-regulated insurance companies and U.K. regulators. The final section provides a conclusion.

## Overview of regulatory and policy developments

This section briefly overviews the major gatekeepers, industry participants and other stakeholders involved in efforts to develop cyber-related regulations and policies that have impacted the U.K. cyber insurance industry over the last decade. These include key central government bodies, cybercrime, cyber monitoring and risk pooling organisations; as well as the key U.K. insurance regulatory bodies, the Bank of England (BofE), the Prudential Regulatory Authority (PRA—a subsidiary of the BofE)—HM Treasury and the Financial Conduct Authority (FCA).^8^ Appendix 1 provides a brief overview of these organisations.

### U.K. government

The U.K. government, mainly through the Home Office and Cabinet Office, has initiated an overall national strategy towards cybersecurity.^9^ This involves the promulgation of government priorities and objectives related to cybersecurity, which involved funding programmes for a U.K. national Cyber Security Programme over five-year periods. The first policy was set out by the Cabinet Office in 2011 and then subsequently updated in 2016 and again in 2022. The first national cybersecurity strategy (U.K. Government 2011) identified four key objectives related to cybersecurity threats through the initiation of a U.K. Cyber Security Programme. This included (1) tackling cybercrime, (2) improving cyber resilience (including information sharing across government departments and industry partners), (3) supporting an open, vibrant and safe cyberspace in the U.K. and (4) building U.K. knowledge, skills and capacity to support U.K. government cybersecurity objectives.^10^ This subsequently resulted in the establishment of a U.K. National Computer Emergency Response Team (CERT-U.K.) in 2014 to strengthen the response to cyber incidents (U.K. Government Cabinet Office 2014).^11^

The strategy was subsequently updated in 2016 when CERT-U.K. was closed and its activities transferred to the National Cyber Security Centre (NCSC), as the U.K. authority responsible for providing leadership on national cybersecurity issues and sharing of knowledge (U.K. Government 2016). Finally, in 2022, the National Strategy was further revised to more specifically focus on detection, investigation and information sharing on state, criminal and other malicious actors to protect the U.K. and to strengthen resilience at the national and organisational levels to prepare for, respond to and recover from cyberattacks (U.K. Government 2022).

### BofE and other financial regulators of cybersecurity

While the U.K. government’s national cybersecurity strategy is maintained at a generic societal level, the BofE has been instrumental in developing specific regulatory guidance to the U.K. insurance industry concerning cybersecurity risk management.^12^ The Financial Policy Committee of the BofE initiated the process in June 2013 by recommending that “HM Treasury, working with the relevant government agencies, the PRA, the Bank and the FCA should collaborate with the financial sector and its infrastructure to put in place a programme of work to improve and test resilience to cyberattack” (BofE 2014). This subsequently resulted in HM Treasury coordinating a work programme, with support from central intelligence agencies, to better understand the threat and strengthen assessment testing and information sharing, focusing on cyber threats affecting financial stability (rather than low-level crime or fraud). It subsequently initiated an independent, intelligence-led vulnerability testing framework (CBEST) to undertake penetration testing of “financial system firms” (BofE 2015b).

Subsequently, the BofE, in conjunction with HM Treasury, set out guidance concerning the evaluation of cyber resilience for general insurers in 2016 (BofE 2016). The BofE, FCA and PRA subsequently issued a joint discussion paper concerning undertaking a “dialogue” with the financial services industry concerning expectations of the regulators and the wider public about the operational resilience of U.K. financial services institutions (BofE, FCA and PRA 2018). This was implemented through an “Operational Resilience Policy”, which required U.K. financial sector firms to be “operationally resilient against multiple forms of disruption (including cyber-related attacks) to minimise the harm caused to consumers and markets (BofE, FCA and PRA 2021a, b, c).^13^

Separately, the PRA issued more specific issues and policy statements concerning its expectations around the prudent management of cyber insurance underwriting risk (PRA 2017a, b). This statement explains the PRA requirements that companies identified quantify and manage cyber exposure and establish risk appetite statements and provide management with exposure metrics. The scope of the statement also includes “non-affirmative” policies (i.e. insurance policies that do not explicitly include or exclude cyber risk coverage, wording exclusions, and attach specific limits to the coverage provided by such policies).

In 2019, the U.K. financial authorities, together with HM Treasury, established a jointly owned “Authorities Response Framework” as a formal way for U.K. financial authorities to coordinate with each other when an incident or threat arises that could cause a major disruption to the U.K. financial services sector.^14^

Finally, in 2021, the PRA issued an operational resilience Statement of Policy. This clarified that all banks and insurers must be operationally resilient through prevention, adaptation and recovery mechanisms (PRA 2021b). Although it did not specifically mention cyber risk sources of disruption, the Policy Statement required that regulated firms connect operational resilience with their governance, operational risk policy business continuity planning and outsourcing activities.^15^ Additionally, the PRA (2021e) issued an implementation guide to provide banks and insurers participating in the CBEST intelligence-led penetrating testing with an updated framework. The purpose of the framework was to help deal with cyber risk as an “important element of operational risk”.

### City of London Police

The City of London Police (CofLP) has been instrumental in developing and coordinating regulatory frameworks against cybercrime, including for the U.K. financial sector. Since 2014, it has hosted the national fraud reporting centre, including cybercrime (CofLP 2014). Subsequently, in 2017, it set up an initiative (Cyber Griffin) to specifically help protect businesses and individuals located in London’s Square Mile from cyberattacks (CofLP 2017). Since 2022, the CofLP has also been responsible for the formation of a national cyber resilience centre group, to strengthen the reach of cyber resilience across U.K. business, and for the Fraud and Cyber Crime Reporting and Analysis System (FCCRAS), to improve the flow of crime, information and intelligence reports through the U.K. national cybercrime ecosystem (CofLP 2022).

### Pool Re

Besides the U.K. regulatory authorities, Pool Re also influences the coordination of cyber risk sharing in the U.K. insurance industry, through the reinsurance of certain types of cyber risk.^16^

Although providing “all risks” reinsurance cover to members for terrorist attacks, Pool Re included acts of cyber terrorism in its coverage in 2018. This coverage is limited to damage caused by terrorists via remote digital interference (Pool Re 2018). It was subsequently extended in 2019 to include non-damage business interruption (Pool Re 2019).^17^ However, the effectiveness of this reinsurance coverage was subsequently called into question when in August 2022 Lloyds of London issued a “market bulletin”, which requires its underwriters to include policy clauses that specifically exclude liability for “losses arising from any state backed cyberattack” (Lloyds 2022). To the extent that state-based attacks are interlinked with terrorist-based cyberattacks, this exemption requirement renders cyber-related risk pooling provided by Pool Re ineffective.^18^

## U.K. policy and regulatory initiatives

This section addresses the second research question by evaluating the scale and scope of the evolution of policy and strategy initiatives that have been undertaken at the national U.K. level by the U.K. government and financial sector and other regulators, as overviewed in the previous section. It then outlines the evolution of a broader set of publicly disclosed collaborative initiatives related to cyber risk that have been undertaken by U.K. regulators together with various other organisations and industry associations.

### Evaluating the quality of U.K. cyber policy coordination

The framework used for evaluating the quality of U.K. cyber policy coordination efforts over the period 2014–2022 is based on the extent to which each of these actions addressed the elements of two frameworks of cybersecurity risk management policy coordination: (1) the nine OECD (2022) general and operational principles for digital security risk management (summarised in Appendix 2) and (2) the BSA (2015) software alliance 2015 European Union cybersecurity maturity dashboard, which identified a number of questions related to: (a) legal foundations, (b) operational entities, (c) public–private partnerships, (d) sector-specific cybersecurity plans and (e) education. Appendix 3 summarises the dashboard element. It also maps the nine OECD (2022) general and operational principles to each question, and additionally reports the BSA (2015) assessment of the extent to which cybersecurity coordination in the U.K. addressed these questions, as of the end of 2014.^19^

Compared to the other 27 EU countries, BSA (2015) identified the U.K. as having a relatively “comprehensive cybersecurity strategy and legal framework”. However, it also showed that, as of the end of 2014, there was a lack of U.K. industry-based risk assessments, and an absence of any legislation or policy that required either annual mandatory reporting of cybersecurity incidents or annual cybersecurity audits (BSA 2015).

Table 1 summarises the timeline of key U.K. policy and regulatory initiatives over the last decade, as outlined in the previous section, when categorised based on the BSA (2015) questions for each of the five major areas summarised above.

**Table 1:** Timeline of key events related to U.K. cyber risk management coordination: regulatory policy and recommendations 2011–2022

Category	Year	Regulator	Summary	Consequence
Legal foundations (1)	2011	U.K. Cabinet Office (2011)	Set out a number of government priorities and objectives related to cybersecurity, including making the U.K. more resilient to cyberattacks and better able to protect U.K. interests in cyberspace. Initiated U.K. National Cyber Security Programme	Maintain and strengthen ability to anticipate, prepare for and disrupt hostile acts in cyberspace (including improving information sharing across government and industry partners, enhancing defence against hostile acts, and increasing law enforcement capability to investigate and prosecute those carrying out hostile acts)
	2016	HM Treasury and HM Cabinet Office	National Cyber Security Strategy 2016–2021	Updated 2011 Strategy by creating a National Cyber Security Centre (NCSC), to be the authority on the U.K.’s cybersecurity environment, through sharing knowledge, addressing systemic vulnerabilities and providing leadership on key national cybersecurity issues
	2022	Cabinet Office	National Cyber Strategy (revised)	Pillar 5. Detect, investigate and share information on state, criminal and other malicious cyber actors and activities in order to protect the U.K., including to strengthen resilience at national and organisational levels to prepare for, respond to and recover from cyberattacks
Operational entities (1)	2016		Creation of National Security Centre	CERT-U.K. was closed and its activities transferred to the NCSC as the U.K. authority responsible for providing leadership on national cybersecurity issues and sharing of knowledge (U.K. Government 2016)
Operational entities (3)	2020	CofLP	National cybercrime portfolio transferred to CofLP	
	2021	Cabinet Office	Creation of National Cyber Force	Combines resources from the Government Communications Headquarter, the Ministry of Defence, the Secret Intelligence Service and the Defence Science and Technology laboratory under a single command, to protect and promote the U.K.’s interests in cyberspace
Operational entities (4)	2014	CofLP	Action Fraud, within the National Fraud Intelligence Burean, becomes part of CofLP	Action Fraud is the U.K.’s national fraud reporting centre, including cybercrime
	2022	Home Office	Creation of Fraud and Cyber Crime Reporting and Analysis System (FCCRAS)	CofLP Commissioner appointed as senior responsible owner for the FCCRAS. To improve the flow of crime, information and intelligence reports through the national cybercrime ecosystem
Operational entities (5)	2014	BofE	Development of intelligence-led vulnerability testing framework (CBEST)	U.K. financial authorities launch CBEST, a testing framework to help firms’ infrastructure providers and regulators understand types of cyberattack that could undermine U.K. financial stability
Operational entities (6)	2019	HM Treasury, BofE (including PRA) and FCA	Authorities Response Framework (ARF)	A formal way for U.K. financial authorities to coordinate with each other. It is used when there is an incident or threat that could cause a major disruption to financial services. The framework is jointly owned, governed, and supported by senior representation from all three authorities. The framework enables the authorities to work together to respond to an incident, whilst ensuring they consider any impacts to their own statutory objectives. All three authorities have a role to play in maintaining the ARF. HM Treasury is the Lead Government Department for the finance sector, which owns the framework, and the BofE has a delegated responsibility to maintain the framework and ensure it remains fit for purpose
Public–private partnerships (1)	2021	NCSC, BofE, PRA, CFA, U.K. Finance	Creation of financial sector cyber collaboration centre	Identify, investigate and coordinate response to cyber incidents that have potential consequences for the U.K. financial sector by combining, analysing and distributing information
	2022	CofLP	Formation of National Cyber Resilience Centre group	A not-for-profit company funded and supported by the Home Office to strengthen the reach of cyber resilience across the U.K. business community
Sector-specific cybersecurity plans (1)	2012–3	BofE (FPC)	Recommended that HM Treasury, relevant government agency, PRA and FCA work with core U.K. financial system and its infrastructure to improve and test resilience to cyberattacks	HM Treasury coordinated work programme across financial authorities with support from GCHQ and Centre for Protection of National Infrastructure
	2016	PRA	Issues Policy Statement PS15/17 Cyber Insurance Underwriting Risk and Supervisory Statement SS 4/17	Sets out PRA expectations regarding the “prudent management of cyber insurance underwriting risk”
	2018	BofE, FCA and PRA	Discussion Paper Building the U.K. financial sector’s operational resilience | Bank of England DP01/18 | Prudential Regulation Authority (PRA) DP01/18 | Financial Conduct Authority (FCA) DP18/04	Seeks to commence a dialogue with the financial services industry on achieving a step change in the operational resilience of firms and FMIs. It aims to generate debate about the expectations regulators and the wider public might have of the operational resilience of U.K. financial services institutions
Sector-specific cybersecurity plans (2)	2016	BofE, HM Treasury, NCSC, FCA	Development of collaborative and coordinated strategy for cyber resilience	Evaluation of cyber resilience at general insurers, subsequently extended to cover other financial services in 2017 (unspecified)
	2018	BofE	Cyber Action Plan	Sets out expectations for financial firms in terms of resilience, testing and arrangements for responding to cyberattacks
	2019	FCA and PRA	Cyber Triage Questionnaire	Collect data to identify and prioritise potential areas for future regulatory focus, including remediation activity, and to support firm- and sector-level analysis
	2019	BofE and FCA	Discussion paper: Building operational resilience: impact tolerances for important business services and feedback to DP18/04	Contains proposals for firms to identify business services at risk of cyberattack, identify and document key people, set impact tolerances
	2021	BofE, FCA and PRA	Operational Resilience Policy PS21/2	U.K. financial sector firms are required to be operationally resilient against multiple forms of disruption to minimise the harm caused to consumers and markets
Sector-specific cybersecurity plans (3)	2015	FPC, BofE	Recommended that BofE, PRA and FCA work with U.K. financial system firms to complete CBEST tests and adopt resilience plans	PRA, FCA CBEST assessments, surveys
	2020	FCA and PRA	Cyber questionnaire (CQUEST)	Developed a cyber questionnaire (CQUEST) jointly with the FCA. Pilot deployment across six banks and seven insurers
Education (1)	2017	CofLP	Creation of Cyber Griffin	An initiative to support businesses and individuals located in the Square Mile to protect themselves from cybercrime

With regard to “legal foundations”, Table 1 shows that the U.K. government has continued to update its national cybersecurity strategy since 2014 (question 1). However, the author was unable to validate evidence to support the BSA (2015) assessment that there was U.K. legislation/policy concerning either a critical infrastructure protection strategy (question 3) or legislative requirements for an information security plan, an inventory of systems and the mapping of security practices to risk levels (questions 4 to 6).^20^

The U.K. shows greater alignment with the “operational entities” questions. Since 2014, there have been new developments concerning the various operational entities that are responsible for implementing the U.K.’s cybersecurity and regulatory policies (questions 1 and 3 to 6).

However, there is limited alignment of the U.K. with the “public–private partnerships” questions. The author could only identify the CofLP’s development of the National Cyber Resilience Centre in 2022 as aligning with question 1. By contrast, there have been a number of U.K. policy and regulatory developments since 2014 related to the “sector-specific cybersecurity plans” area, while the creation of “CyberGriffen” by the CofLP in 2017 addresses the “education” area.

Moreover, there has not been any subsequent efforts made by U.K. regulatory authorities since 2014 to address legal foundation questions related to annual cybersecurity audits (legal foundations, question 7) and imposing mandatory public disclosure of cyber breaches (question 10).

### Broader cyber-related U.K. collaborations^21^

The U.K. Home Office and Marsh (2015) provided an early report on the role of the U.K. insurance industry in establishing cyber risk insurance for U.K. firms, and the role of London as a global centre for cyber risk management. It recommended that Lloyds, the Association of British Insurers (ABI) and the U.K. government develop more guidance for the cyber insurance industry, and facilitate the development of a cyber risk pool by the U.K. insurance sector. Additionally, it recommended that Lloyds and the U.K. Department for Trade and Investment (UKTI) cooperate to promote cyber insurance offerings of the London market to key countries around the world.

The BofE has also addressed broader collaboration issues concerning cybersecurity, both nationally and internationally. The Bank of England Quarterly Bulletin published an article by Warren et al. (2018), which identified links between cyber risk and systemic risk. It highlighted the developing coordination of the NCSC, U.K. Finance and U.K. Financial Authorities to analyse and distribute information concerning cyberattacks and increase the financial sector’s resilience to cyber threats. Subsequently, these organisations collaborated to create a U.K. “Financial Sector Collaboration Centre” to identify, investigate and coordinate the response to cyber incidents affecting the U.K. financial sector (NCSC 2021).^22^

The BofE has also been active in publicising its ongoing work on coordinating cybersecurity in the U.K. financial sector at industry conferences in 2015 (BofE 2015b) and 2021 (BofE 2021b, 2021c).

Finally, Pool Re has also been active in publicising cyber terrorism threats. In collaboration with the Cambridge Centre for Risk Studies (CCRS), it sponsored a report assessing the threat of cyber terrorism and proposed a variety of cyber terrorism attack scenarios potentially affecting vulnerable sectors that could comprise its membership (Evan et al. 2017). The report concluded that while most relevant cyber terrorist actors pose a low likelihood of inflicting severe damage, greater monitoring of such threats was needed.^23^

However, U.K. regulatory authorities have also received criticism. In its 2021 financial system stability assessment report on the U.K., the IMF (2022) recommended that U.K. regulatory authorities (specifically the BofE, FCA and PRA) should “enhance cyber risk technical risk reviews on technology risk management expectations for all financial firms, and by conducting additional cybersecurity control verification activities to complement CBEST security testing” (Recommendation B5). Only the PRA and FCA publicly responded to the findings of the 2021 IMF assessment report but did not specifically respond to this recommendation (PRA 2022; FCA 2022).^24^

## International collaboration efforts

The OECD’s digital security risk management principles include encouraging collaboration across borders (OECD 2022, general principle 4). This section briefly overviews international collaboration efforts related to cybersecurity management coordination, some of which involve U.K. regulatory authorities.

At the international level, the BofE has been involved in establishing cooperation for identifying and responding to global cybersecurity threats. Table 2 summarises the major developments.

**Table 2:** Timeline of key events related to U.K. cyber risk management: international collaboration, surveys and projects 2011–2022

Year	Authority Regulator(s)	Summary	Consequence
2015	HM Government, Cabinet Office and Marsh	Reports how insurance can help make U.K. companies more resilient to cyber threats	Provides recommendations to help firms understand cyber risks, help the insurance industry to establish cyber insurance and to help London become a global centre for cyber risk management
2015	Pool Re	Established the International Forum of Terrorism Risk (Re) Insurance Pools	Undertakes annual conferences on the urgent challenges concerning prospective protection gaps, advances in actions to assess gaps, how best to integrate risk management strategies between public sector agencies, how to harness reinsurance capabilities to meet UN sustainable development goals, how to build resilience to cyber terrorism as an emerging risk and how to adapt to the blurred lines between terrorism and war in the cyber context. Resulted in subsequent joint working party with The Geneva Association
2016	G7	Creation of cyber expert group	Joint coordination exercises, publication of fundamental elements of cybersecurity (2016), effective assessment of cybersecurity (2017), third-party cyber risk management (2018) and cyber exercise programmes (2020)
2017	Pool Re and University of Cambridge, Centre for Risk Studies	Assess the threat of cyber terrorism to the U.K.	Proposes a variety of cyber terrorism attack scenarios which could affect vulnerable U.K. industry sectors that compromise the exposure of the Pool Re membership
2018	EU-U.S. Dialogue Project	Insurance industry cybersecurity issues paper	Outlines existing legislative and supervisory frameworks in the EU and U.S. and describes selected initiatives and resources
2018	BofE Quarterly Bulletin	Could a cyberattack have a systemic impact on the financial sector?	Concludes that there is a credible case to link cyber risk to systemic risk in the financial sector. Makes various recommendations
2019	FCA	Cyber and technology resilience: themes from a cross-sector survey	Identifies governance and cyber resilience as a top concern and that there is scope for improving information sharing and IT change management functions and identifyng challenges in managing third parties
2019	BofE, PRA and Monetary Authority of Singapore	Collaborate to strengthen cybersecurity in financial sectors in the U.K. and Singapore	Identifies effective ways to share information and explores potential for staff exchanges
2020	U.K. and EU	U.K. leaves EU	End of coordination of U.K. and EU cybersecurity risk management
2020– 2022	The Geneva Association and IFTIP	Three publications: (1) Cyber War and Terrorism (2020); Mapping a Path to Cyber Attribution Consensus (2021) and (3) Insuring Hostile Cyber Activity (2022)	(1) Introduces HCA as a potential tool for the industry to bridge the gap between terrorism and war (2) Promotes a recognised, industry-wide approach to attribution, or identifying the responsible actor. Proposes a series of steps and checklists to structure the process of attribution and charaterisation for insurers. Emphasises importance of building collaboration across insurance, technology and government to develop an international norm to promote a consistent and streamlined approach for attribution (3) Ultimately, a form of government backstop or PPP is needed to finance extreme cyber risks. Designing such a solution is complex as it will involve trade offs in adopting particular scheme features and difficulties calibrating how much of the peak losses should be shared among policyholders, private re/insurers and governments

In 2016, the BofE cochaired a G7 expert group on cyber with the U.S. Treasury, resulting in a statement about identifying fundamental elements of cybersecurity for the financial sector (G7 2016a, b, c). This subsequently resulted in the G7 publishing further statements concerning fundamental elements for (i) effective assessment of cybersecurity (G7 2018), (ii) third-party cyber risk management in the financial sector (G7 2016a, b, c), (iii) threat-led penetration testing and (iv) cyber exercise programmes (G7 2020).^25^

Pool Re has also been active globally and initiated the International Forum of Terrorism Risk (Re)Insurance Pools (IFTRIP) in 2015, which was ratified at a conference organised by the Australian Reinsurance Pool Corporation in 2016. Its members and observers include reinsurance organisations based in 10 countries, including Austria, Denmark, France, Germany, the Netherlands and Spain. ITRIP has subsequently held three further annual conferences to facilitate the exchange of information concerning mitigation and capacity building against economic losses arising from terrorism. IFTRIP subsequently produced three joint reports with The Geneva Association on cyber terrorism-related topics concerning the definition of hostile cyber activity (HCA) (The Geneva Association 2020), providing a framework for the attribution and characterisation of cyber incidents (The Geneva Association 2021) and the ability of private re/insurers to underwrite HCA risks and how public–private partnerships can provide more effective solutions (The Geneva Association 2022).

More generally, the PRA (2016, 2017a, b, 2019, 2020, 2021a) has stated that its functions are aligned with the International Association of Insurance Supervisors’ (or “Basel”) core principles regarding a Common Framework for the Supervision of Internationally Active Insurance Groups (IAIS 2019). These core principles include “The supervisor requires insurers and intermediaries to have policies and processes for the protection and use of information on customers”, which include “assessing the potential impact of new and emerging risks that could threaten the privacy of personal information, such as the risk of cyberattacks” (IAIS 2019, Recommendation 19.12).

The Financial Stability Board (2022) has proposed a common format for cyber incident reporting, including the development of a common format for incident reporting exchange. The consultation document included a survey of initial reporting trigger reference material in 17 jurisdictions, including the U.K.. Unlike incident reporting requirements in most other countries, the relevant PRA material (FCA Rule Book, SUP 15.3 General Notification Requirements) does not make specific reference to cyberattacks. It also does not prescribe a specified time deadline for reporting when such incidents have taken place.^26^

U.K. regulatory authorities also participate in bilateral cyber risk assessments. For example, the IMF (2017) reported in its 2016 financial stability assessment that the U.S. and U.K. authorities conducted a joint exercise with major global financial firms in November 2015 to enhance their cooperation and ability to respond to cyberattacks. However, none of the three U.K. financial sector authorities disclosed this exercise in their annual reports.

Other international initiatives related to cybersecurity coordination do not involve the participation of U.K. regulatory authorities, following the U.K.’s departure from the European Union in 2020 (EU 2019).^27^ For example, the EU-U.S. Insurance Dialogue Project includes a “cyber insurance market working group” which aims to pursue “an ongoing bilateral dialogue to share knowledge and experiences with respect to the cyber insurance market”. U.K.-registered insurance companies are also no longer subject to EIOPA supervisory guidelines related to various cyber issues, such as the recently produced statements concerning “*management of non-affirmative cyber exposures”* (EIOPA 2022a) and *“exclusions in insurance products related to risks arising from systemic events* (EIOPA 2022b).^28^ Furthermore, the CofLP no larger participates in various EU-led initiatives combating cybercrime established by EUROPOL, such as the European Cybercrime Centre (ECC) established in 2013, the Internet Organised Crime Threat Assessment (IOCTA) and the Joint Cybercrime Action Taskforce.

## Evaluating the effectiveness of cyber risk coordination in the U.K.

This section addresses the third research question by employing a multi-method approach to evaluate the effectiveness of the cyber risk coordination efforts in the U.K. insurance cyber underwriting industry over the last decade, as outlined in the previous section.^29^ Three dimensions considered in this section comprise an analysis of trends over the last decade concerning the impact of cyber coordination on the evolution of (1) the estimated total direct costs associated with the incidence of cyberattacks in the U.K. financial sector during 2014–2022; (2) the evolution and nature of investment in computer software and system intangible assets that are subject to a cyberattack; (3) a content analysis of the incidence of “cyber” and cyber-related (non-named noun) term citations in the annual reports of (a) a sample of five large U.K.-registered insurance companies which either do or do not provide cyber insurance policies^30^; and (b) the three main U.K. financial regulatory authorities (i.e. the BofE, FCA and PRA)^31^; and (4) an assessment of the degree to which regulated U.K. insurance companies have enhanced their operational resilience to cyberattacks over time.

### Total costs of cyber incidents affecting the U.K. financial sector

This section provides an estimate of the evolution of the total cost of cyber-related incidents in the U.K. financial sector and investments in cybersecurity programmes made by U.K. financial regulators over the past decade. Three primary information sources were used to make these estimates. First, the FCA disclosures of the total number of reported incidents (including cyber-related) in its annual reports. Second, the Ponemon Institute’s annual reports of the total costs of data breaches (in USD million). Third, the FCA’s reports on the total investments in progress related to cybersecurity programmes in the footnotes to its financial statements contained in its annual reports.^32^

**Fig. 1:**
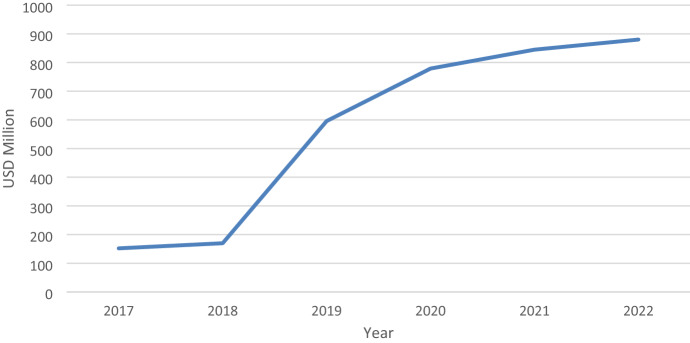
Estimated total cost of data breaches, U.K. financial sector 2017–2022 (USD million)

In order to estimate the total estimated costs of reported cyber incidents in the U.K. financial sector, the total number of such incidents reported annually to the FCA is multiplied by the cost of a data breach as disclosed in the annually updated Ponemon Institute (2013, 2014, 2015, 2016, 2017, 2018, 2019, 2020, 2021) survey report.^33^ Figure 1 reports the evolution of the total estimated costs associated with reported cyber incidents related to the U.K. financial sector over the five years since the FCA first publicly disclosed the number of incidents reported.^34^

Figure 1 shows an eight-fold increase in the total cost of data breaches in the U.K. financial sector, rising from an estimated USD 150 million in 2017 to USD 875 million in 2022. This was based on an increase in the cost per data breach from USD 152 million in 2017 to USD 880 in 2022, and a five-fold increase in the total number of cyber incidents reported to the FCA from 42 incidents in 2017 to an estimated 202 incidents in 2022.

### Investment in computer software and systems

**Fig. 2:**
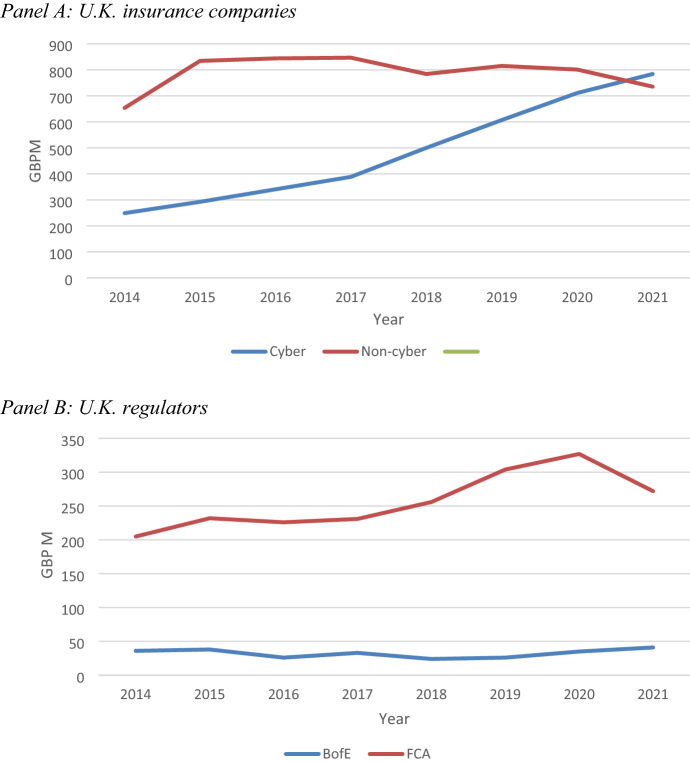
Value at risk—total cost of U.K. computer systems and software investments 2013–2021

IAS 38, Intangibles (IFRS 2022) permits reporting entities to recognise certain types of intangible assets, which are defined as “an identifiable non-monetary asset without physical substance” (IAS 38, paragraph 8).^35^ Most U.K. financial sector regulators and insurance companies recognise their investment in computer systems and software as intangible assets.^36^ This provides valuable information concerning the total value at risk of such assets, which are the subject of cyberattacks.^37^ Figure 2 reports the total value (in GBP million) of investment in computer software and systems intangible assets by the sample of U.K.-based insurance companies (Panel A) and U.K. financial regulatory authorities (Panel B), over the study period 2014–2021.^38^

Panel A shows that the total value of investment in intangible assets related to computer systems and software by the sample of U.K. cyber insurance firms has increased two fold over the study period. By contrast, the equivalent investments by the sample of non-cyber U.K. insurance firms initially increased, but then declined consistently over time. Further, their total investments in 2021 were slightly lower than for the sample cyber insurance firms (i.e. GBP 714 million versus GBP 735 million).^39^

Panel B of Fig. 2 shows contrasting trends over the study period in the total amount invested in computer systems and software intangible assets for the two U.K. financial regulators. The average FCA investments are also eight times larger than those of the BofE.^40^ While the total investment in IT systems increased by 33% over the entire study period, it declined by 17% from 2020 to 2021. By contrast, the total investment in computer software and systems intangible assets by the BofE remained relatively constant over the entire study period (increasing by 14%).

**Fig. 3:**
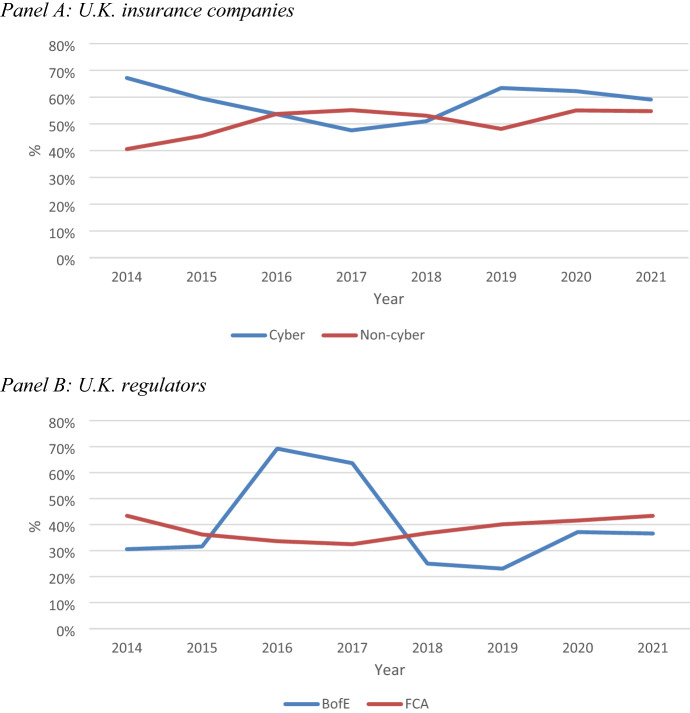
Net-to-gross book value of software and computer system investments 2014–2021

Further insights into the nature of these investments can be revealed by the extent to which the sample U.K. life insurance firms and U.K. financial regulators have recorded accumulated amortisation on these investments.^41^ These accounting estimates provide valuable information on the extent to which these reporting entities are relying on legacy systems, which presumably are potentially more susceptible to cyberattacks. Figure 3 shows the percentage of “net” (i.e. after subtracting total accumulated amortisation from the total historical cost of investment) to “gross” values of intangible assets related to computer software and systems for the sample of U.K. insurance companies (Panel A) and financial regulators (Panel B).^42^

Panel A of Fig. 3 shows that the average percentage of net-to-gross carrying amount of intangible assets related to computer systems and software for the sample of U.K. insurance firms ranged from 58% (cyber) to 51% (non-cyber). However, there are considerable variations over time, with the percentage declining slightly over the entire period for cyber firms by 12%, while increasing by 35% for non-cyber firms. This suggests that non-cyber insurance firms’ investment in computer software and systems is relatively more at risk from cyberattacks than for cyber insurance firms, but not significantly.

By contrast, Panel B shows that both U.K. financial regulators have a relatively lower percentage of net-to-gross average book value over the study period (40% for BofE and 38% for FCA). While this has increased over time for the BofE (20%), it decreased significantly after 2017.^43^ By contrast, there was no change in the equivalent trend for the FCA.

### Content analysis of annual reports

**Fig. 4:**
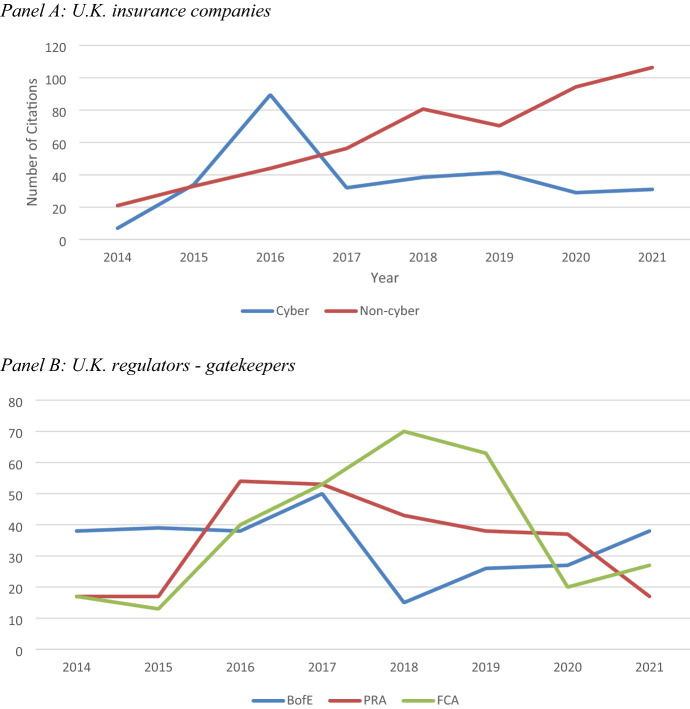
Content analysis of annual reports: citations of “cyber” related terms 2014–2021

In order to establish the nature and intensity of engagement with cybersecurity by the sample insurance companies and regulators, a researcher-designed content analysis was employed to analyse the frequency of the citation of the non-noun word “cyber” in annual reports of major insurance and reinsurance companies and key U.K. regulators for the period 2014–2021. Additionally, 19 non-named noun words associated with cyber (e.g. “denial of service” and “crime”) were also included in the content analysis. Table 3 reports the list of all 20 terms included in the content analysis. Figure 4 reports the trends in the total number of citations of “cyber” and cyber-related terms that were cited in the annual reports produced by the sample cyber and non-cyber U.K. insurance companies (Panel A) and the three U.K. regulatory authorities (Panel B) over the study period.

**Table 3:** Content analysis of annual reports

Nr	Term (non-named noun)
1	Cyber
2	Attack
3	Breach
4	Crime
5	Data fraud, data theft, data loss
6	Denial of service
7	Disruption
8	Failure
9	Hack
10	Incident
11	Malware
12	Malicious
13	Phishing
14	Ransomware
15	Resilience
16	Risk
17	Scam
18	Security
19	Threat
20	Virus

List of terms searched (related to cyber, IT and/or computer)

Panel A of Fig. 4 reveals inconsistent patterns in the evolution of the citation of “cyber” for the sample cyber and non-cyber U.K. firms over time. The average number of citations for non-cyber firms increased significantly over the period (over four times) and was significantly higher than for non-cyber firms (63 vs 38). By contrast, the number of citations for cyber firms increased significantly from 2014 to 2016, but declined thereafter. This suggests that regulatory efforts to coordinate cybersecurity management in the U.K. insurance sector appears to have had only transitory effects on the extent to which cyber insurance firms recognise cybersecurity as an issue.

Panel B of Fig. 3 shows that the average number of citations of the three U.K. regulatory authorities is quite similar (34 for BofE and PRA, and 38 for the FCA). However, there are significant variations in the number of citations over time. The BofE annual report citations of cyber-related terms increased initially from 2014 to 2017, but then declined significantly in the following three years, before rising slightly in 2021. The PRA citations showed a similar trend, but in contrast declined significantly in 2021. Conversely, trends in the pattern of the number of citations of “cyber” in annual reports of the FCA increased significantly from 2015 to 2018, but then declined significantly in the following two years, before rising slightly in 2021.

The overall conclusion of this section is that there is only limited evidence concerning the effectiveness of U.K. regulatory authorities’ efforts to coordinate cyber risk management in the U.K. insurance sector. On the one hand, there has been a significant increase in the total cost of cyberattacks in the U.K. financial sector, and the level of investment by non-cyber insurance firms in intangible assets related to computer systems and software intangible assets has also increased, both in total in the percentage of net-to-gross book carrying values. These firms have also increased their recognition of cyber and cyber-related terms in their annual reports over time. The content analysis also revealed that only one non-cyber insurance company provided voluntary disclosure of the nature and incidence of cyber-related events on its insurance business in 2021. This disclosure is reproduced in Appendix 4.

On the other hand, there is only limited evidence that cyber is being recognised by cyber insurance firms. While the total amount of their investment in computer software and systems has increased significantly, the percentage of net-to-gross book values of these investments has declined, therefore increasing the vulnerability of these firms to cyberattacks. Furthermore, the content analysis revealed that their recognition of cyber-related terms declined over time.

By contrast, there are inconsistent patterns in the total investments in IT by the two U.K. regulators. While the level of investment is significantly higher for the FCA than the BofE, the percentage of net-to-gross book values of these investments has declined significantly, while that of the BofE has remained relatively constant. However, both authorities have a significantly lower level of net-to-gross book value of these investments than the insurance firms, indicating that they are relying more on “legacy systems” that are more susceptible to cyberattacks and IT failure more generally, as the PRA (but not the BofE or FCA) admitted occurred in September 2016 (PRA 2017a, b). Moreover, there have been inconsistent and declining citations of cyber-related terms in their annual reports over the study period, indicating that they do not see the significant increases in data breach costs associated with cyber as a matter of increasing accountability, to their key stakeholders and to society in general.

Furthermore, only the FCA has provided disclosure of its investment in cybersecurity software and systems as part of its “work in progress” intangible assets in 2021. “*£1.8 m relates to Cyber Security Programme to reduce cyber security risks and both consumer and firm harm through embedding appropriate controls, technology, processes and behaviours across the FCA”* (FCA 2018, footnote 8, p. 163).

Finally, although there is no specific regulatory requirement in the U.K. for (insurance) companies to report how they manage their cyber risks, provision 31 of the U.K. Corporate Governance Code (Financial Reporting Council 2018) requires company directors to assess the future viability (prospects) of the company strategy and risk appetite, by reference to the principal risks faced, and how these are managed. Only one U.K. non cyber insurance firm (effective from annual reporting year 2016) consistently lists IT and cyber-related operational risks as a principal risk to its business, which it defines as: “…the risk of loss (or unintended gain or profit) arising from inadequate or failed internal processes, personnel, and systems, or from external events. This includes employee error, model error, system failures, fraud or some other event which disrupts business processes” (Prudential plc 2016, p. 58).

## Conclusion

This paper provides evidence on the nature and evolution of the regulatory coordination of cybersecurity risk management in the U.K. cyber insurance industry in a period of increasing cyber threats and rising global tensions related to state-based and terrorist cyberattacks. It first provides a descriptive overview of the historical evolution of both regulatory interventions and then overviews broader national and international collaboration efforts with other organisations, associations and industry bodies. These are categorised in terms of both the BSA’s cybersecurity maturity dashboard and the OECD’s framework for cybersecurity (BSA 2015; OECD 2022). Multiple research methods are then used to evaluate the effectiveness of these developments, comprising an estimate of the evolution of the cost of cyberattacks, estimates of the total and net book value investment in computer systems and software intangible assets, and a content analysis of cyber-related terms cited in annual reports of a sample of (cyber and non-cyber) U.K. insurance companies and U.K. regulatory authorities.

The major findings are summarised below:

- The estimated cost of data breaches has significantly increased over time. The incidence of systemic, state-based attacks has also increased, as evidenced most recently by the recent attack on the Danish financial authorities and financial sector firms. It has also resulted in cyber underwriters strengthening their exclusion clauses to exclude state-based cyberattacks.
- In response to these threats, the U.K. government has regularly updated its national cyber strategy. Furthermore, there have been a number of domestic initiatives undertaken since 2014 by the U.K. financial regulatory authorities, including expanding the scale and scope of their cyber-related initiatives and policies, initiating an authorities response framework to facilitate coordination, setting up new private partnerships, and encouraging regulatees to strengthen their operational resilience to deal with such threats. Additionally the CofLP has taken central responsibility in the fight against cybercrime.
- Internationally, U.K. regulatory authorities have also initiated collaborative coordination efforts, through setting up cyber expert group organisations such as the G7, the creation of IFRIC by Pool Re, as well as participating in joint exercises with other countries, both via the G7 and bilaterally.
- There remain a number of regulatory gaps and overlaps. There is no single financial regulatory authority that has responsibility for the supervision of insurance firms, unlike the situation in the EU (EIOPA). Furthermore, there remain gaps in the maturity of U.K. cybersecurity coordination efforts, as initially identified by the BSA (2015), related to mandating public disclosure of cyberattacks and the imposition of annual cybersecurity audits.
- Brexit has ended the previous collaboration between U.K. and EU police authorities in fighting cybercrime, and the applicability to U.K. insurance firms of subsequent regulatory guidance provided by EIOPA concerning cybersecurity risk management of the European insurance industry, which has not since been provided by U.K. regulatory authorities. This has consequently increased the susceptibility of both U.K. financial regulatory authorities and U.K. cyber insurance firms to systemic state and-or terrorism related cyberattacks.
- Despite the rising costs of estimated data breaches during 2014–2021, the investment in computer system-related intangible assets by U.K. regulatory authorities has not significantly increased, while their level of net-to-book value has declined. This increases their potential susceptibility to systemic state- and/or terrorism-based cyberattacks.
- Compared to non-cyber-underwriting U.K. insurance firms, there is a relatively lower level of transparency by both key cyber underwriting U.K. insurance firms and U.K. regulatory authorities, both in relation to the total average number of citations of cyber-related terms and trends over time. More generally, there is a lack of transparency concerning the nature and extent of reporting of cyber-related incidents by these entities. Moreover, there is no specific U.K. regulatory requirements for the reporting of cyber incidents.

It is concluded that, while the U.K. regulatory authorities appear to have been relatively successful in preventing wide-scale and systemic cyberattacks on the U.K. insurance industry in recent years, their regulatory and policy actions have not resulted in significantly enhanced operational resilience by U.K. cyber insurance-regulated firms. Furthermore, the recent decision by Lloyds to require its syndicates to specifically exclude terrorist-related cyberattacks from their cyber insurance and reinsurance policies raises questions about the effectiveness of Pool Re’s decision to provide such insurance cover to its members.

Moreover, the research has also uncovered apparent gaps and overlaps in regulatory oversight, with the development of regulatory monitoring mechanisms of industry-specific responses to cyber threats by the BofE, PRA and FCA taking place apparently independently and separately from cybercrime oversight by the CofLP and relevant national U.K. cyber authorities. Additionally, despite U.K. government initiatives to develop and evolve a national cyber policy, there is a lack of focus of regulatory efforts on the U.K. cyber insurance industry specifically. There are also inconsistencies in enforcement powers between different U.K. regulators in relation to cybersecurity risk-related policies. Contrary to both the sweeping criminal law powers of the CofLP and the specific legislative enforcement mechanisms available to the FCA, there is a lack of enforcement of the powers of the BofE and its subsidiary PRA, with regulatory guidance restricted to relatively “soft touch” principles-based guidelines and policies.

These findings are subject to a number of caveats. First, the quality of the research undertaken is limited by a lack of transparency concerning cyberattacks by relevant U.K. regulatory authorities and sample U.K. insurance firms. In contrast to the situation in other countries such as the U.S., there are no mandatory disclosure requirements imposed on either U.K. insurance companies or regulatory authorities concerning the incidence and/or the cost of cyber-related data breaches. The lack of publicly available information concerning the incidence and nature of IT system failure and data breaches thereby affects the ability of the research to draw evidential-based definitive conclusions as to the operational resilience of both U.K. regulators and insurance company regulatees to withstand systemic cyberattacks of the kind that have most recently occurred in Denmark.

Second, the BSA (2015) and OECD (2022) frameworks for categorising types of cybersecurity policies and practices are not compatible in many aspects, so there is a significant degree of subjectivity in categorising the extent and nature of various U.K. regulatory policy initiatives based on a common framework of analysis. Furthermore, the quality of data sources that are available to estimate the costs of data breaches is not of consistent quality over time, with the Ponemon Institute providing only high-level, generic costs of data breaches. Finally, the content analysis is based on a relatively subjective, researcher-defined content analysis index and is therefore subject to alternative interpretations and definitions.

Further research can be undertaken to extend the analysis and findings of this paper in a number of directions. Firstly, international comparisons of both national and global efforts to coordinate cyber-related risk management in the insurance sector would provide more insight into the relative effectiveness of various regulatory regimes. Second, the content analysis of annual reports can be extended both in terms of highlighting specific aspects of cyber risk management systems that are best practice, as well as comparing the impact of alternative regulatory regimes on the degree and nature of cyber risk management engagement by insurance companies in the U.K. and internationally. Further research is also needed to identify best practice reporting of cyber resilience, in order to both reduce information asymmetry among investors and to enhance societal confidence in the cybersecurity-related operational resilience of the insurance industry globally.

Finally, given the importance of the global insurance industry in both underwriting cyber risk and managing its own exposure to cyberattacks, further collaborative, policy-level efforts are needed to develop a publicly available database of both the cost and nature of incidents at the national and international levels. This will in turn help to facilitate greater public awareness of the relevant issues and thereby encourage more informed and rigorous academic research into this increasingly important topic.

## Data Availability

Data is not publicly available in relation this article.

## Appendix 1: Overview of U.K. regulatory bodies related to cybersecurity

### Bank of England (BofE)

The BofE is the central bank of the U.K.. Following the passage of the Financial Services Act (2012), an independent financial policy committee was created. The BofE was also authorised to establish a new prudential regulator, the Prudential Regulation Authority (see below).

### City of London Police (CofLP)

The CofLP, formed in 1832, is responsible for the policing of the city of London. It was granted national authority for fighting cybercrime in 2018. It also hosts the National Fraud Intelligence Bureau (NFIB), now established as the U.K.’s central fraud and cybercrime intelligence hub.

### Financial Conduct Authority (FCA)

The FCA was created in April 2013 under the Financial Services Act 2012 and the Financial Services (Banking Reform) Act 2013 to take over certain conduct and relevant prudential regulation from the former Financial Services Authority (FSA). It regulates the conduct of 50,000 financial firms in the U.K..

### National Cyber Security Centre (NCSC)

The NCSC is an organisation of the U.K. government established in 2017. It is responsible for responding to cybersecurity incidents and seeks to reduce cyber risks to the U.K. by securing public and private sector networks. It also plays a role in disseminating cybersecurity guidance.

### Pool Re

Pool Re is a reinsurer corporation that operates as a public–private partnership to reinsure property damage and business interruption against terrorism. It was created by the U.K. insurance industry in cooperation with the U.K. government in 1993 following the IRA bombing campaign on the U.K. mainland. Membership is open to any U.K. insurer authorised to insure losses arising from damage to commercial property. Subsequent to the September 11 attacks in 2001, its cover was extended to an “all risks” basis. Claims are paid only when HM Treasury issues a certificate when a particular event is deemed an act of terrorism.

### Prudential Regulatory Authority

The PRA, a subsidiary of the BofE, is responsible for the prudential supervision of 1,500 financial institutions in the U.K., including banks and insurance companies.

### U.K. Government

The U.K. Government has initiated various cybersecurity strategies. The Home Office is the “corporate headquarters” of the U.K. Government, in partnership with HM Treasury. It takes the lead in several critical policy areas, such as cybersecurity strategy.

## Appendix 2: OECD Policy Framework on Digital Security Risk Management General Principles (OECD 2022)

**Table:**

Nr	Category	Principle topic	Description
1	General	Digital security culture: Awareness, skills and empowerment	All stakeholders should create a culture of digital security based on the understanding of digital security risk and how to manage it
2		Responsibility and liability	All stakeholders should take responsibility for the management of digital security risk based on their roles, the context and their ability to act
3		Human rights and fundamental values	All stakeholders should manage digital security risk in a transparent manner and consistently with human rights and fundamental values
4		Cooperation	All stakeholders should cooperate, including across borders
5	Operational	Strategy and governance	Leaders and decision makers should ensure that digital security risk is integrated in their overall risk management strategy and managed as a strategic risk requiring operational measures
6		Risk assessment and treatment	Leaders and decision makers should ensure that digital security risk is treated on the basis of continuous risk assessment
7		Security measures	Leaders and decision makers should ensure that security measures are appropriate to and commensurate with the risk
8		Innovation	Leaders and decision makers should ensure that innovation is considered
9		Resilience, preparedness and continuity	Leaders and decision makers should ensure that a preparedness and continuity plan based on digital security risk assessment is adopted, implemented and tested to ensure resilience

## Appendix 3: European Union Cybersecurity Maturity Dashboard BSA (2015)

**Table:**

OECD principle	#	Question	U.K. Evaluation (end of 2014)
Legal foundations			
5	1	Is there a national cybersecurity strategy in place?	Yes
-	2	What year was the national cybersecurity strategy adopted?	2011
5	3	Is there a critical infrastructure protection (CIP) strategy or plan in place?	Yes
2	4	Is there legislation/policy that requires the establishment of a written information security plan?	Partial
6	5	Is there legislation/policy that requires an inventory of “systems” and the classification of data?	Yes
7	6	Is there legislation/policy that requires security practices/requirements to be mapped to risk levels?	Yes
1	7	Is there legislation/policy that requires (at least) an annual cybersecurity audit?	No
1	8	Is there legislation/policy that requires a public report on cybersecurity capacity for the government?	Partial
5	9	Is there legislation/policy that requires each agency to have a chief information officer (CIO) or chief security officer (CSO)?	No
3	10	Is there legislation/policy that requires mandatory reporting of cybersecurity incidents?	No
2	11	Does legislation/policy include an appropriate definition for “CIP”?	Yes
8	12	Are requirements for public and private procurement of cybersecurity solutions based on international accreditation or certification schemes, without additional local requirements?	Partial
Operational entities			
9	1	Is there a national computer emergency response team (CERT) or computer security incident response team (CSIRT)?	Yes
-	2	What year was the CERT established?	2014
5	3	Is there a national competent authority for network and information security (NIS)?	Yes
6	4	Is there an incident reporting platform for collecting cybersecurity incident data?	Yes
7	5	Are national cybersecurity exercises conducted?	Yes
9	6	Is there a national incident management structure (NIMS) for responding to cybersecurity incidents?	Yes
Public–private partnerships			
4	1	Is there a defined public–private partnership (PPP) for cybersecurity?	Yes
4	2	Is there industry organised partnerships (i.e. business or industry cybersecurity councils)?	Yes
4	3	Are new public–private partnerships in planning or underway (if so, which focus area)?	NA
Sector-specific cybersecurity plans			
5	1	Is there a joint public–private sector plan that addresses cybersecurity?	Yes
5	2	Have sector-specific security priorities been defined?	Partial
6	3	Have any sector cybersecurity risk assessments been conducted?	No
Education			
1	1	Is there an education strategy to enhance cybersecurity knowledge and increase cybersecurity awareness of the public from a young age?	Yes

## Appendix 4: Example of data breach disclosure (Prudential 2022, p. 105)

A total of 18 data breaches were reported and collectively involved personal data of 47,266 individuals. The top three types of data breaches were (i) loss of policy documents in transit (33%); (ii) data disclosed to incorrect recipient by email, post, or other means (33%); and (iii) SMSs sent to wrong customers or terminated distribution representatives (22%). Out of the 18 data breaches reported, six involved sensitive health information and collectively impacted 113 individuals. The six data breaches were related to policy document loss, data disclosed to incorrect recipients via post or emails, and a contractor sending unencrypted files to an external email address. While the incidents do not represent any systemic issue, mitigation actions have been taken to prevent recurrence of the incidents.

## Footnotes

1 Cybersecurity is the body of technologies, processes and practices designed to protect networks, systems, computers, programmes and data from attack, damage or unauthorised access. CNI is defined as “certain ‘critical’ elements of infrastructure, the loss or compromise of which would have a major, detrimental impact on the availability or integrity of essential services, leading to severe economic or social consequences or to loss of life”.

2 According to The Financial Times, cybersecurity attacks on companies doubled in 2012–2013 compared with the previous financial year (Financial Times 2015). The Lloyd’s (2016) risk index survey revealed that cybersecurity risk was the third most important perceived risk faced by U.K. business, significantly higher than in 2011 when it was only ranked 12th. Subsequently, Lloyds collaborated with the Cambridge Centre for Risk Studies (Coburn et al. 2014a, b) to develop a city risk index, which showed that cyber risk was one of the most important emerging risks.

3 In the U.S., the Securities and Exchange Commission (SEC) requires registrants to disclose, as part of the management discussion and analysis part of their annual report filing (“10-K”) the risk of cyber incidents if these issues are among the most significant factors that make an investment in the company speculative or risky. However, Ferraro (2014) argues that these SEC disclosure requirements are too vague and not sufficiently informative.

4 The Danish media reported that a number of Danish banks, including the central bank, were subject to cyberattacks during a continuous period from 11 until 15 January 2023 (www.dr.dk/flere). A Russian hacker group “Killnet” claimed responsibility for the attacks, stating that it was a result of Denmark supporting Ukraine in the aftermath of the Russian invasion in early 2022. Unlike the U.K., Denmark does not currently have a national cyber strategy.

5 In March 2013, six South Korean banks were affected by a suspected North Korean cyberattack, disrupting financial services worldwide and costing GBP 500 million to clear up (The Guardian 2013). Subsequently, the percentage of nation-state cyberattacks targeting critical infrastructure has increased from 18% in 2020 to 40% in 2022 (Burt 2022). Most recently, the Danish financial sector was subject to a state-based cyberattack in 2023.

6 The Australian Strategic Policy Institute (2014) proposed greater geopolitical engagement on cyberattacks but did not focus distinctly on the financial system. Aldarsoro et al. (2021) also recommend greater regulatory coherence in financial services but did not refer to cybersecurity issues specifically.

7 This paper addresses Giddens (1999) notion of “risk society”, i.e. that society is increasingly concerned about the future. Emerging risks related to both climate change and cybersecurity are examples of such threats.

8 This section is limited to focusing only on coordination efforts by U.K. national regulatory authorities. Because of the impact of Brexit, it does not include a discussion of European Union-wide regulatory initiatives, e.g. those undertaken by the European Insurance and Occupational Pensions Authority (EIOPA 2018), the European Union Agency for Network and Information Security (ENISA 2016, 2022) and relevant EU Directives (e.g. GDPR).

9 Additionally, the U.K. Department for Business, Innovation and Skills (2014) has initiated a certification scheme for U.K. organisations (Cyber Essentials Scheme) more generally to implement to mitigate cyber risk.

10 The Cabinet Office (2013) updated the original national cybersecurity strategy by reference to BofE initiatives (see below). However, the Intelligence and Security Committee of the House of Commons (2013) raised issues of potential ethical conflict between commercial imperatives and national security as a result of increasing private ownership of telecommunications networks that are considered to be part of the U.K.’s critical national infrastructure.

11 CERT also included the Cyber Security Information Sharing Partnership (CiSO), a scheme that enabled government and industry to share information on a current threat.

12 This section only discusses coordination efforts by U.K. financial regulatory authorities. It does not discuss coordination efforts at the more generic societal level, such as initiatives of the National Cybersecurity Centre or the Home Office.

13 Besides specified regulatory coordination actions, the PRA and FCA also initiated a series of questionnaires, including a “cyber triage questionnaire” concerning financial sector firms’ remediation activity (FCA and PRA 2019).

14 This is a separate initiative from the more generic Cabinet Office Meeting Rooms (COBRA), which refers to the offices used by key central government bodies when more generic threats to the U.K. arise.

15 The PRA also issued specific policies concerning outsourcing and third-party risk management (PRA 2021c), and internal management (PRA 2021d). This is not within the scope of this paper.

16 In an earlier study, Long Finance (2015) recommended a U.K. public–private cyber catastrophe reinsurance scheme to address the systematic nature of cyber risks and the potential for “cyber catastrophes”. It is not known whether these findings facilitated the subsequent decision by Pool Re to incorporate cyber risk coverage into its policies.

17 It should be noted that although the breadth of terrorism covered by Pool Re is “all risks”, excluding war and cyber, cyber terrorism that results in damage is then “added back” (Pool Re 2021).

18 The Geneva Association and IFTRIP (2022) propose a common definition of “hostile cyber activity” but do not clearly delineate state-based from terrorism-based cyberattacks.

19 Since the OECD (2022) general principles and the BSA (2015) were developed independently, the extent to which they can be “mapped” to each other is subject to subjective judgement. The OECD (2022) framework is also more limited in scope, as it focuses on national public policy-level issues.

20 The Critical Infrastructure Information Act (2002) facilitates the sharing of critical infrastructure information among the owners and operators of U.K. critical infrastructure and government entities with infrastructure protection responsibilities, to reduce the U.K.’s vulnerability to terrorist attacks. However, the legislation applies only to physical infrastructure (e.g. energy, telecommunications) and does not cover cyber-related attacks.

21 This section only covers the contributions made by U.K. gatekeepers and financial sector regulatory organisations to public debates and forums concerning cyber risk. Several other organisations have also published contributions, for example on the role of insurance in cybersecurity, e.g. CRO Forum (2014, 2017), OECD (2017), and more generally to reviewing issues and risk management standards in cybersecurity (ENISA 2016, 2018, 2022). Additionally, the IAIS (2016) produced a paper based on a survey of members concerning raising awareness for both insurers and supervisors of current practices and regulatory issues.

22 Additionally, there are also collaboration groups in the U.K. in which the BofE is involved, such as the Cross Market Operational Resilience Group (CMORG) and the Financial Services Information Sharing Analysis Centre (FS-ISAC) (BofE 2021b).

23 The CCRS has produced several important publications related to cybersecurity risk management, including a standard data scheme for managing cyber exposures (CCSC 2016) and a framework for the systematic assessment for macrocatastrophes (Coburn et al. 2014a, b), and a report outlining best practices for developing scenario analysis for the insurance industry (Strong et al. 2020).

24 The previous IMF (2017) financial system stability assessment of the U.K. referred to efforts made by the authorities to identify and respond to cybersecurity as a “top priority” but did not include any specific recommendations.

25 This followed the first cross-border coordination exercise across the G7, involving 23 financial authorities in 2019, including the BofE, PRA and FCA (BofE 2021b).

26 By contrast, the FSB (2022) survey refers to the European Central Bank’s requirements for reporting of such incidents. See the discussion below on EU cybersecurity coordination requirements.

27 The agreement between the U.K. and the EU concerning the U.K.’s departure from the EU (EU 2019) does not specifically mention any ongoing collaboration agreements concerning the coordination of cyber-related issues, either at the national, industry or cybercrime authority level.

28 None of the U.K. regulatory authorities have issued equivalent guidance to U.K.-registered insurance companies.

29 It should be noted that this analysis is not comparable to the “cost benefit analysis” that the FCA and other U.K. regulatory authorities are required to undertake to analyse the impact of their regulatory interventions (e.g. FCA 2018a, b, c, d). Rather, it is meant to provide, based on a combination of quantitative and qualitative methods, key insights as to the degree of influence of various policy and collaborative efforts on cyber risk management in the U.K. insurance industry.

30 The IAIS (2022) produces an annually updated register of internationally active insurance groups (IAIGs), which includes three non-cyber insurance underwriting U.K.-registered insurance companies: Aviva plc (2015, 2016, 2017, 2018, 2019, 2020, 2021, 2022), Legal and General plc and Prudential plc. Additionally, for the purposes of completion of analysis, annual reports issued by two U.K.-based insurance companies that actively provide cyber insurance (Direct Line Group plc 2015, 2016, 2017, 2018, 2019, 2020, 2021, 2022, and Hiscox Ltd, 2015, 2016, 2017, 2018, 2019, 2020, 2021, 2022) have also been included for analysis in this section. However, it is recognised that these insurers are significantly smaller in terms of both size and premiums written. It should also be noted that while Hiscox operates in the U.K., it is registered in Bermuda.

31 The PRA, as a subsidiary of the BofE, does not provide sufficient information in its annual reports (i.e. related to investment in intangible assets in software and computer systems), which are presumably shared with the BofE. The CofLP was excluded, as it does not produce annual reports on a consistent basis over the entire study period, and these do not contain sufficient information related to either cyber or financial information.

32 Eling and Wirfs (2019) point out that information on cyber risk is not publicly available since companies that have suffered security breaches or have been attacked do not report it, and because an authoritative definition of “cyber risk” is lacking.

33 Ponemon Institute annual reports of cyber-related incidents are not consistently available by country and industry sector. Therefore, the average total cost of a data breach for the total number of companies participating in this research is used.

34 The FCA did not report the number of reported cyber-related incidents in its 2019, 2020 and 2021 annual reports. Instead, this was estimated to be the annual average of reported cyber incidents as a proportion of total reported incidents in the previous three years (i.e. 26% of total reported incidents).

35 IAS 38 (paragraph 21) requires that an intangible asset can only be recognised if (a) it is probable that the expected future economic benefits that are attributable to the asset will flow to the entity, and (b) the cost of the asset can be recognised reliably. It is therefore possible that the total value of investments in computer systems and software is considerably higher than that which is reported in this section.

36 As a subsidiary of the BofE, the PRA does not report any computer software as intangible assets. Lloyds’ investment in computer software intangible assets is immaterial and is therefore not included in the analysis. The CofLP does not produce publicly available financial statements.

37 Other types of intangible assets that can be recognised under IAS 38 include intangible assets arising from business combinations (goodwill), customer relationships, brands and trademarks. For the purposes of the analysis, it is assumed that computer systems are the first order value at risk arising from a cyberattack, although it is likely that the impact could be much broader (e.g. company franchise value, reputation), depending on the severity of the cyberattack.

38 These figures are based on the total or gross value of the recognised investment in computer software and systems intangible assets. This differs from its net or “carrying amount”, i.e. the amount at which the intangible assets are recognised in the balance sheet, after deducting any accumulated amortisation and impairment losses, therefore. Amortisation is the systematic allocation of the depreciable amount of an intangible asset over its useful life, which is discussed separately below.

39 These figures are not strictly comparable across the sample of U.K. insurance companies because both Aviva plc and Prudential plc sold and/or disposed of subsidiaries during 2017–2021.

40 This analysis assumes that the two regulatory agencies maintain separate computer systems. However, the FCA (2018a, b, c, d) reported that it continued to share “legacy systems” of the former Financial Services Authority (which was replaced by the FCA and PRA in 2014) with the PRA. The PRA (2017a, 2017b) admitted that there was a “disruption to normal operations during a brief outage which affected certain shared systems in September 2016” but did not provide further details. The FCA (2017) annual report did not reveal that this incident had occurred.

41 Alternatively, the net carrying amount of the total investment in computer software and equipment could be reported in this section. While this net carrying amount is more likely to reflect the approximate value in accounting terms, the gross carrying amount is reported here as the “replacement cost” is more relevant.

42 Caution should be taken when interpreting these figures since there are considerable variations in the depreciation rate assumptions both across the sample firms and for different types of computer systems and software, which can range from two to seven years depending on the scale and nature of the investment.

43 This could be due to the “lumpy” nature of investments in computer software and equipment on a year-by-year basis.
